# Listerial Spontaneous Bacterial Peritonitis

**DOI:** 10.7759/cureus.22051

**Published:** 2022-02-09

**Authors:** Samira Samant, Bradley Uyemura, Pandit Sarbagya, Pinky Jha

**Affiliations:** 1 Internal Medicine, Medical College of Wisconsin, Milwaukee, USA

**Keywords:** spontaneous bacterial peritonitis, listeria monocytogenes, ascites, listeria sbp, corynebacterium

## Abstract

Spontaneous bacterial peritonitis (SBP) is a severe complication of ascites often seen in advanced hepatic disease that is most commonly caused by Gram-negative bacilli. Here, we report a rare case of *Listeria monocytogenes* SBP, diagnosed by peritoneal fluid culture and responsive to ampicillin, in a patient with portal hypertension secondary to nodular regenerative hyperplasia. Because Listeria species are resistant to empiric SBP therapies and delays in treatment have been associated with increased mortality, they must be considered in high-risk patients.

## Introduction

Spontaneous bacterial peritonitis (SBP) is an infection of the ascitic fluid that arises without a definitive intra-abdominal source and is most commonly seen in advanced hepatic disease [1]. Crucial to the development of this infection is bacterial translocation; that is, the failure of containment of bacteria within the bowel that allows the passage of bacteria from the intestinal lumen to mesenteric lymph nodes, which subsequently affords the possibility of spread to the bloodstream or other extra-intestinal sites [2]. This translocation may be preceded by bacterial overgrowth associated with an impaired intestinal barrier, diminished immunologic defenses, or decreased bowel motility in cirrhotic patients. While some cases of SBP are associated with the development of acute septic shock, others may be asymptomatic; the latter is often identified when a patient with cirrhosis undergoes routine paracentesis [1].

A diagnosis of SBP must be made in the absence of any identifiable etiology of infection or secondary bacterial peritonitis, including gastrointestinal tract perforations, diverticulitis, cholecystitis, or appendicitis, and requires a sample of ascitic fluid [3]. A polymorphonuclear (PMN) count greater than 250 cells/mm^3^ suggests SBP, and a positive ascitic fluid culture typically confirms the diagnosis [3].

The most common causative organisms in SBP tend to be Gram-negative bacilli; the three most common are *Escherichia coli*, Streptococcus, and Klebsiella species, although numerous other bacteria have been demonstrated to cause SBP at a much lower incidence [3]. Antibiotic treatment for SBP is designed with the causative predominance of coliform bacteria in mind. Third-generation cephalosporins are typically the antibiotics of choice, as they have a broad antibacterial coverage coupled with a good safety profile; cefotaxime is the most commonly used agent in this class [2]. Oral fluoroquinolones may also be used in patients not suffering from nausea or emesis, or in patients allergic to cephalosporins.

While a short or long course of antibiotics is typically sufficient to resolve the infection, occasionally these measures prove ineffective. In such cases, it is crucial to investigate further, as a less frequently implicated bacterium may be the underlying cause of SBP.

## Case presentation

Our patient was a 71-year-old female with a past medical history significant for hypertension, Crohn's disease, and non-cirrhotic portal hypertension with esophageal varices and ascites secondary to nodular regenerative hyperplasia, who presented with a chief complaint of rectal pain and abdominal distension. Her home medications were carvedilol, furosemide, spironolactone, infliximab, and famotidine. Her allergy history was significant for reactions to sulfa drugs and augmentin.

**Figure 1:**
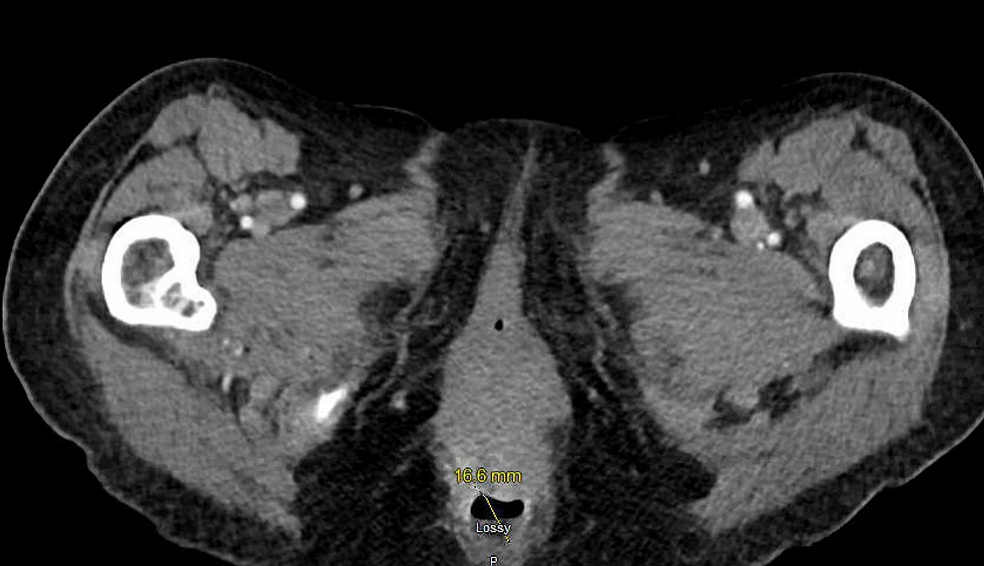
Perianal abscess identified on CT A/P

One month prior to this admission, she had been evaluated for a fever of unknown origin, with an extensive workup significant for a CT abdomen and pelvis showing colitis. While antibiotics proved ineffective, prednisone abated her fever. Shortly after steroid treatment, she developed worsening abdominal distension. Paracentesis removed 5.5 L of cloudy-yellow fluid containing an elevated white blood cell count of over 5000 cells/mm^3^, with 86% PMNs. She was started on a ten-day course of cephalexin but nine days later was admitted for severe rectal pain and abdominal distension. Repeat paracentesis yielded 600 ml of cloudy-yellow fluid with a white blood cell count of 2800 cells/mm^3^, with 82% PMNs. Her white count was elevated from 7.6 × 103/µL to 17 × 103/µL, but BMP and transaminases were unremarkable. A CT abdomen and pelvis revealed cirrhotic changes, new left-sided pleural effusion, increased ascites, and a new 2.5 cm focus of air-fluid level contiguous with the posterior anus and extending to the gluteal cleft, suspicious for a perianal abscess (Figure 1). Biopsy of her liver revealed a few foci of alternating compressed and normal liver cell plates, suggestive of nodular regenerative hyperplasia; minimal foci of sinusoidal fibrosis; and minimal portal inflammation primarily consisting of mononuclear cells. Her ascitic fluid yielded Gram-positive rods, and the culture grew cephalexin-resistant Corynebacterium. The cephalexin was switched to vancomycin for more appropriate antimicrobial coverage, but she developed diarrhea. When trended, her improving erythrocyte sedimentation rate and C-reactive protein made a Crohn's flare unlikely. A *C. difficile* nucleic acid amplification test was positive, but she was found to be toxin-negative, suggesting antibiotic-induced diarrhea.

Given her recent CT A/P findings, infectious disease suspected the Corynebacterium was a contaminant from her perianal abscess; reevaluation of her ascitic culture revealed *Listeria monocytogenes*. A further, more detailed history-taking revealed that she owned a dairy farm, and her husband had recently suffered from a gastrointestinal illness. She likely contracted enteric Listeria that seeded her peritoneal fluid. Ampicillin was initiated, and repeat paracentesis showed a recovering white cell count, with negative Gram stain and culture. She was discharged on amoxicillin.

## Discussion

Listeria is a Gram-positive rod that can be regularly isolated from soil, water, vegetation, and even asymptomatic humans [4]. Though rare cases of human infections by *L. lvanovii* and *L. grayi* have been reported, *L. monocytogenes* is the primary pathogen in humans [5,6]. Given that *L. monocytogenes* can thrive in a wide pH range and refrigerator temperatures, it has been associated with a variety of foodborne outbreaks including processed meats, soft cheeses, and raw produce [5,7-10].

Listeriosis typically manifests as self-limiting febrile gastroenteritis [11]. While less than 1% of reported cases of bacterial foodborne infections are attributed to Listeria, it can cause invasive diseases such as meningitis, meningoencephalitis, and bacteremia in susceptible individuals, with mortality rates as high as 20-30% [12,13]. Populations that have been shown to be predisposed to Listeria infection include neonates, pregnant women, the elderly, and the immunocompromised [14-17]. Our patient had been receiving glucocorticoid therapy for the prior two months for her Crohn's disease prior to the transition to infliximab; notably, glucocorticoids have also been specifically associated with an increased risk of Listeriosis [18]. In addition, it has been shown that farm animals represent a reservoir for human *L. monocytogenes* infections [19].

Listeria was first described as a cause of SBP in 1977 [20], but has remained exceptionally rare. A literature review published in 2015 listed just 106 reported cases of Listeria SBP, with only 11 cases in the United States [21]. Instead, nearly 40% of cases have been reported in Spain, despite the fact that human listeriosis, in general, is not preferentially elevated in the region [22,23]. Though it is unclear why SBP secondary to Listeria is predominantly reported in Spain, it is speculated that cultural differences involving increased consumption of contaminated dairy products, produce, and processed meats could be contributing.

Empiric therapy for SBP generally involves third-generation cephalosporins, such as cefotaxime or ceftriaxone, given their historically good coverage of the most common pathogens, which include *E. coli*, *Klebsiella pneumoniae*, and pneumococci [24,25]. However, several reports have demonstrated failure rates of empiric cephalosporin treatment as high as 43% [26,27]. In fact, *L. monocytogenes* has consistently been reported to be intrinsically resistant to third-generation cephalosporins due to poor cephalosporin target-binding [28]. As such, ampicillin with an aminoglycoside, if the toxicity profile allows, has been successfully used to treat SBP caused by *L. monocytogenes* [29]. Though the associated symptoms of Listeria peritonitis are largely indistinguishable from any other causes of SBP, delayed treatment from initial incorrect empiric antibiotic therapy has been associated with worse outcomes, with an overall mortality of 30% [23]. Thus, prompt evaluation of risk factors, geography, occupational exposures, and recent foodborne illnesses/outbreaks can be essential for suspecting Listeriosis and consequently adjusting empiric antibiotic therapy for *L. monocytogenes* coverage.

Because the cumulative recurrence rate at one year of SBP is nearly 70%, prophylactic antibiotics are indicated [30]. While norfloxacin is typically used for SBP prophylaxis, it often does not provide coverage for *L. monocytogenes*. As such, amoxicillin or trimethoprim-sulfamethoxazole is recommended for patients recovering from Listeria SBP [31].

## Conclusions

Listeria is an exceptionally rare cause of SBP. Its presentation closely mimics that of other pathogens, making the recognition of ascitic or blood cultures with Gram-positive rods crucial, along with the recognition of risk factors such as immunosuppression, exposure to farm animals or contaminated foods, and iron overload. Listeria must be considered as a potential causative organism in patients with SBP who are not responding to conventional therapy, especially if such risk factors are present. Because it is resistant to some cephalosporins, ampicillin - with an aminoglycoside if tolerated - is the treatment of choice. Following an initial episode of Listeria SBP, the recurrence rate is as high as 70% in one year; for this reason, prophylactic antibiotics are typically initiated as a preventative measure. Listeria frequently has innate fluoroquinolone resistance, making trimethoprim-sulfamethoxazole the prophylactic antibiotic of choice.
